# Macrophage-derived FGF21 Restrains Endometritis By Suppressing Inflammation and Promoting Repair Through FGFR1

**DOI:** 10.1007/s10753-026-02537-z

**Published:** 2026-07-21

**Authors:** Haiyan Liu, Zhuo Shi, Yihui Zheng, Xianliang Chen, Fatma Mrisho Haji, Weiwei Zhu, Feng Lin

**Affiliations:** 1 Department of Gynecology, the First Affiliated Hospital of Wenzhou Medical University, Wenzhou, 325035 Zhejiang China; 2 The Second Affiliated Hospital and Yuying Children’s Hospital of Wenzhou Medical University, Wenzhou, 325035 Zhejiang China; 3 Department of Cardiology, the First Affiliated Hospital of Wenzhou Medical University, Wenzhou, 325035 Zhejiang China

**Keywords:** FGF21, Endometritis, Anti-inflammatory, Tissue Repair

## Abstract

**Abstract:**

Endometritis is a prevalent inflammatory disorder that impairs female fertility, yet effective therapeutic strategies remain limited. Although the fibroblast growth factor (FGF) family has been increasingly implicated in inflammatory diseases, its role in endometritis remains largely unexplored. Here, systematic profiling of the FGF family identified FGF21 as the most significantly upregulated member in inflamed uterine tissues. Mechanistically, FGF21 is predominantly derived from macrophages and is positively associated with inflammatory activation. Functional studies showed that genetic deletion of FGF21 markedly exacerbates uterine inflammation, whereas exogenous administration of recombinant human FGF21 (rhFGF21) alleviates tissue injury and suppresses inflammatory responses in lipopolysaccharide-induced endometritis. At the cellular level, FGF21 attenuates macrophage-mediated inflammation and promotes proliferation while inhibiting cell death in endometrial stromal cells. Importantly, these protective effects are recapitulated in a clinically relevant methicillin-resistant *Staphylococcus aureus*-induced model, where rhFGF21 exhibits robust dose-dependent therapeutic efficacy. Mechanistically, FGF21 exerts its effects through activation of fibroblast growth factor receptor 1 (FGFR1), and pharmacological inhibition of FGFR1 abolishes its anti-inflammatory and pro-repair functions. Collectively, our findings identify macrophage-derived FGF21 as a critical regulator of endometrial inflammation and tissue repair, and highlight the rhFGF21 as a potential therapeutic drug for endometritis.

**Graphical Abstract:**

**Figure:**
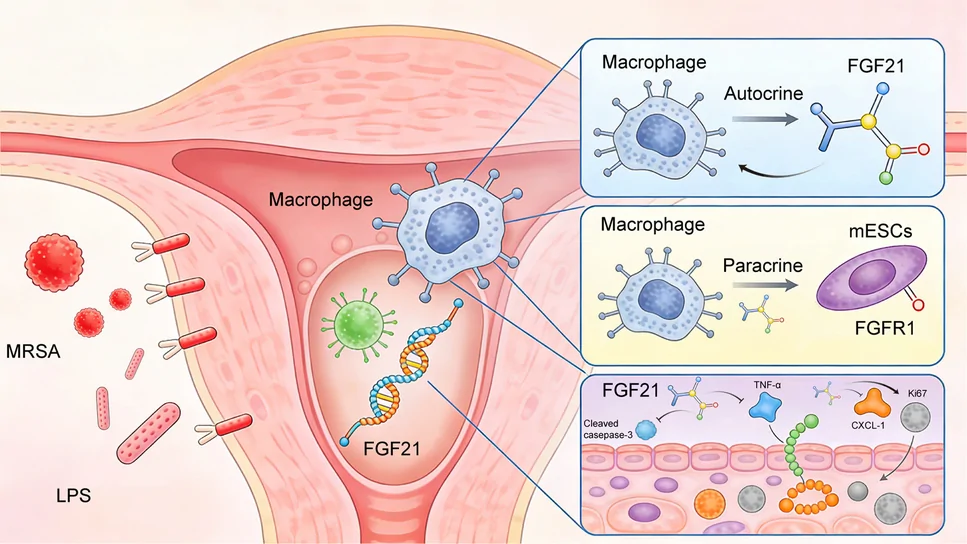

**Supplementary Information:**

The online version contains supplementary material available at https://doi.org/10.1007/s10753-026-02537-z.

## Introduction

Endometritis, a common inflammatory disorder of the uterine lining [1], is primarily associated with microbial infections, including bacteria such as Escherichia coli, Streptococcus, and Staphylococcus aureus, as well as other microorganisms including Mycoplasma species [2]. This condition can manifest as acute symptoms such as pelvic pain and abnormal uterine bleeding, and in its chronic form is strongly associated with infertility, recurrent pregnancy loss [3, 4], and implantation failure in assisted reproductive technologies (ART) [5, 6], thereby posing a significant threat to female reproductive health [7]. The current clinical management of endometritis relies heavily on antibiotic therapy [8, 9]. Although antibiotics can effectively resolve infection and improve reproductive outcomes in some cases, a substantial proportion of patients exhibit persistent endometrial inflammation despite standard treatment [10]. Therefore, given the growing challenge of antibiotic resistance and the limited efficacy of conventional therapies in modulating dysregulated immune responses and promoting tissue repair, there is an urgent need to develop effective therapeutic strategies to improve fertility in patients with endometritis.

In recent years, fibroblast growth factors (FGFs), a family of secreted proteins comprising 22 members, have attracted increasing attention due to their diverse roles in development, metabolism, tissue repair, and immune regulation [11, 12]. Emerging evidence indicates that multiple FGF family members are actively involved in the pathogenesis of inflammatory diseases, where they regulate immune cell activation, cytokine production, and tissue remodeling. In addition, FGFs have been implicated in various uterine and reproductive processes, including endometrial regeneration, embryo implantation, and uterine remodeling. Despite these advances, the potential involvement and functional significance of FGF family members in endometritis, particularly in the context of local inflammatory responses within the uterine microenvironment, remain largely unclear, especially with respect to their cell-specific sources and mechanisms of action.

As an endocrine member of the FGF family, fibroblast growth factor 21 (FGF21) is predominantly expressed in the liver and functions as a key regulator of systemic metabolism [13]. Beyond its metabolic roles, accumulating evidence has demonstrated that FGF21 exerts significant anti-inflammatory and organ-protective effects in various pathological conditions, including systemic inflammation [14], cardiovascular diseases [15, 16], and non-alcoholic steatohepatitis [17–20]. Notably, FGF21-deficient mice exhibit increased mortality following lipopolysaccharide (LPS) challenge, supporting a protective role for FGF21 in inflammatory stress [21]. However, these studies have largely focused on metabolic organs and systemic responses, while the expression pattern, cellular source, and functional role of FGF21 in local reproductive tract inflammation remain poorly defined.

In current study, to systematically explore the potential involvement of the FGF family in endometritis, we performed a comprehensive expression screening of FGF members in a mouse model of endometritis. Among all FGF family members, FGF21 was identified as one of the most prominently altered factors. Further analysis revealed that FGF21 is primarily secreted by macrophages within endometrial tissues. Based on these findings, we next investigated the functional role and underlying mechanisms of FGF21 in endometritis. Using a combination of FGF21 knockout mice, exogenous recombinant protein supplementation, cell co-culture systems, and molecular and cellular approaches, we aimed to: (1) define the functional role of FGF21 by evaluating disease severity in FGF21-deficient mice and assessing the therapeutic potential of exogenous FGF21 in established preclinical models; and (2) delineate the molecular mechanisms underlying FGF21 action, with a particular focus on identifying its functional receptor and validating its requirement in mediating anti-inflammatory effects. Collectively, this study provides new insights into the pathogenesis of endometritis and offers a potential therapeutic strategy for improving reproductive outcomes.

## Materials and Methods

### Ethics Statement

All animals in this study received humane care in accordance with the criteria outlined in the Guide for the Care and Use of Laboratory Animals. All animal protocols were approved by the Laboratory Animal Center of the First Affiliated Hospital of Wenzhou Medical University, Zhejiang, China (Ethics No. WYYY-AEC-2024–004).

### Chemicals and Reagents

Dulbecco's modified Eagle's medium (DMEM), RPMI-1640, penicillin, and streptomycin were purchased from Gibco (Grand Island, USA). Bovine serum albumin (BSA) was obtained from Energy Chemical (Anhui, China). Recombinant human FGF21 (rhFGF21, CM110) and Macrophage Colony-Stimulating Factor (M-CSF, CM061) were purchased from Chamot Biotechnology Co., Ltd. (Shanghai, China). PD173074 was purchased from GLPBIO (Montclair, USA). Soluble starch was obtained from Solarbio (Beijing, China). Beef extract was purchased from Macklin (Shanghai, China), and tryptone was obtained from Oxoid (Beijing, China). DNase I and collagenase I were purchased from Biosharp (Beijing, China).

### Cell Culture

Mouse primary peritoneal macrophages (MPMs) were prepared as previously described [22]. Briefly, C57BL/6 mice (6–8 weeks old) were intraperitoneally injected with 2 mL of 6% thioglycolate solution (0.3 g beef extract, 1 g tryptone, and 0.5 g sodium chloride dissolved in 100 mL double-distilled water and filtered through a 0.22 μm filter). After 3 days, peritoneal exudate cells were collected and centrifuged to obtain MPMs. Cells were cultured in RPMI-1640 medium supplemented with 10% fetal bovine serum (FBS) and 1% penicillin/streptomycin at 37 °C in a humidified 5% CO₂ atmosphere. After 6 h, non-adherent cells were removed by washing with phosphate-buffered saline (PBS), and adherent cells were further cultured in fresh medium. Cells were used for subsequent experiments after 24 h. Cellular morphology was observed using an optical microscope.

RAW264.7 cells (ATCC, TIB-71™) were cultured in DMEM (high glucose) supplemented with 10% FBS and 1% penicillin/streptomycin.

Primary endometrial epithelial cells (EECs) and endometrial stromal cells (ESCs) were isolated from adult (8–10 weeks old) female C57BL/6 mouse uteri and cultured as previously described [23, 24]. Briefly, uteri from non-pregnant mice were dissected, minced, and digested with 0.25% trypsin/EDTA at 4 °C for 2 h followed by 37 °C for 30 min. The cell suspension was filtered through a 70-μm nylon mesh to obtain EECs, which were cultured in DMEM/F12 medium containing 10% FBS and antibiotics. The remaining tissue was further digested with collagenase I at 37 °C for 1 h to obtain ESCs, followed by filtration through a 40-μm mesh and centrifugation. Cells were cultured and medium was changed every 1–2 days until ~ 90% confluence. Cell purity (> 95%) was confirmed by immunofluorescence staining of cytokeratin 18 (EECs) and vimentin (ESCs).

The extraction of murine bone marrow-derived macrophages (BMDMs) were performed using a method adapted from published approaches [25, 26]. BMDMs were extracted from the femurs and tibias of C57BL/6 mice (6–8 weeks old). Briefly, after cervical dislocation of mice, bilateral femurs and tibias were immediately isolated and placed in HBSS buffer. Both ends of the femurs and tibias were cut off, and the marrow cells were flushed out using a 5 mL syringe with HBSS buffer, repeating the process three times for each femur and tibia. After centrifuging at 800 × g for 5 min, RBCs were lysed using RBC lysis buffer. The cell suspension was filtered through a 40 µm cell strainer, centrifuged again to obtain the cell pellets, and then resuspended in DMEM culture medium containing 20 ng/mL M-CSF and 10% FBS before being seeded into cell culture flasks. Three days later, the medium was completely replaced to remove non-adherent cells, and subsequently, half of the medium was changed every 3 days.

### Mice Models and Treatment

Female C57BL/6 wild-type mice (8–10 weeks old, 20–25 g) were purchased from Zhejiang Vital River Laboratory Animal Technology Co., Ltd. Female FGF21 knockout (KO) mice were provided by Professor Jingling Shen’s research group. Mice were housed under controlled conditions (12 h light/dark cycle) with free access to food and water.

Endometritis was induced by intrauterine perfusion of 10 μL LPS (5 mg/mL) [27, 28] or 10 μL *Staphylococcus aureus* suspension (OD = 0.95) [29] into each uterine horn. Recombinant human FGF21 (rhFGF21) was administered intraperitoneally for 3 consecutive days prior to LPS challenge. One day after the final injection, LPS was administered intrauterinely. Mice were sacrificed 48 h after modeling, and uterine tissues were either fixed in paraformaldehyde or stored at − 80 °C for further analysis.

C57BL/6 wild-type female mice were divided into the following groups: (1) sham-operated group; (2) LPS-induced and 0.9% saline injected group; (3) LPS-induced and rhFGF21 (1.5 mg/kg)-injected intraperitoneally group; (4) MRSA-induced and 0.9% saline injected group; (5) MRSA-induced and rhFGF21 (0.75 mg/kg)-injected intraperitoneally group; (6) MRSA-induced and rhFGF21 (1.5 mg/kg)-injected intraperitoneally group. Female FGF21 KO mice were divided into two groups:(1) sham-operated group;(2) LPS-induced and 0.9% saline injected group. To evaluate potential effects of rhFGF21 on normal uterine tissue, a group of healthy wild‑type female mice was treated intraperitoneally with rhFGF21 (1.5 mg/kg) for three consecutive days (rhFGF21‑alone group). The rhFGF21 was dissolved in phosphate-buffered saline (PBS), and the dose of 0.75 mg/kg and 1.5 mg/kg rhFGF21 was intraperitoneally injected into the mice three days in advance [30].

### Scratch Test

Cells were seeded into a 6-well plate. When cells adhered to wall, they were cultured by serum-free DMEM. When cell confluence reached 90%−100%, a 10 μL-pipette was used to perpendicularly and slowly scratch on the bottom of the culture plate, and 4–5 wounds with the same width were created in each well. The cells were washed with PBS three times to remove scratched cells. Subsequently, corresponding experimental treatments were carried out and then incubated in incubator. After 0,12 and 24 h of scratch, inverted microscope was performed to observe the migration distance of cells, and cells were photographed under several randomly-selected fields. This experiment was repeated three times.

### EDU Staining

Primary endometrial stromal cells (ESCs) were seeded into 24-well flat-bottom plates, and stain the cells when the cell density reaches 80% (n = 3). The cells were sequentially processed through EDU labeling (Beyotime, C0075S, China), fixation, washing, permeabilization, and counterstaining with Hoechst 33,342. Following these steps, a fluorescence microscope (Olympus, FV4000) was used to capture images and quantify the proportion of cells labeled with EDU.

### TUNEL Assay for Apoptosis Detection

Primary endometrial stromal cells (80–90% confluence) were seeded into a 24-well plate and incubated at 37 °C for 24 h. After incubation, the cells were washed twice with PBS. The control and treated groups were fixed with 4% paraformaldehyde for 30 min, followed by permeabilization with 0.3% Triton X-100 for 15 min. The cells were then stained with TUNEL reagent for 60 min and DAPI for 10 min to stain the nuclei. Finally, the cells were washed twice with PBS. TUNEL Apoptosis Detection Kit (GLPBIO, GK10040, China). The apoptotic cells were observed under a fluorescence microscope (Olympus, FV4000).

### HE Staining and Histological Inflammation Score

Uteri samples were fixed in 4% buffered para formaldehyde for 48 h, dehydrated in increasing ethanol graduations, diaphanized in xylol, and then embedded in paraffin blocks. Histological Sects. (4 μm thick) were stained with hematoxylin–eosin (H&E) for standard histological analysis and toluidine blue for mast cell assessment. Histopathologic scoring of the mammary gland was performed as previously described [31].The major histopathological indicators were evaluated by hyperemia (graded 0–3, from normal to severe, including normal, mild, moderate, and severe) and infiltration with neutrophils (graded 0–5, 0, from absent to transmural).

### Immunohistochemistry

Samples were fixed with 4% formaldehyde, embedded with paraffin, cut into 4 μm serial sections and then placed in an incubator at 60 °C for 1 h. The sections were dewaxed with xylene, dehydrated with gradient ethanol, and incubated in 3% H_2_O_2_ (Sigma-Aldrich Chemical Company, St. Louis, MO, USA) for 30 min at 37 °C. Then, sections were washed with phosphate buffered saline (PBS), boiled in 0.01 mol/L citric acid buffer for 20 min at 95 °C, washed with PBS after cooled to room temperature, and blocked with normal goat serum working solution for 10 min at 37 °C. Subsequently, sections were added with 50 μL primary antibody (including the following antibodies: FGF21 (bsm-52978R, 1:200), IL-1β (AF5103, 1:200), TNF-α (AF7014, 1:200), F4/80 (28,463–1-AP, 1:500), Ki67 (AF0198, 1:200), Cleaved-Caspase 3 (AF7022, 1:200), p-FGFR1(AF3157, 1:200)) at 4 °C overnight and washed with PBS, added with the horse radish peroxidase (HRP)-conjugated IgG second antibody and incubated at 37 °C for 30 min. The sections were rewashed with PBS three times with 3 min each time, and developed with diaminobenzidine (Sigma-Aldrich Chemical Company). After that, the sections were re-stained with hematoxylin (Bogoo Biotechnology, Shanghai, China) and then mounted with neutral resin. The sections were observed and photographed under optical microscope (XSP-36, Shenzhen Bo as of Optical Instrument Co., Ltd., Shenzhen, China) and five high power fields (HPF) of each section were randomly selected.

### Immunofluorescence Assay

Cells and tissue sections were permeabilized with 0.1% Triton X- 100, exposed to blocking solution (10% BSA) and incubated with primary antibodies at 4 ◦ C overnight. The cells and tissue sections were then incubated with fluorescently labelled secondary antibodies for 1 h at room temperature. The cell nuclei were stained with DAPI, and fluorescence images were captured under a fluorescence microscope (Olympus, Japan).

### ELISA for Cytokines Examination

Uterine tissues were weighted and homogenized with cold PBS on ice. The homogenates were collected and centrifuged at 12,000 g for 15 min at 4℃. The supernatants were collected to detect the concentration of TNF α and IL-1β by ELISA kits according to the manufacturer’s instructions, including mouse IL-1β (#88–7013-77), and mouse TNF-α (#88–7324-76).

### RNA Extraction and qRT-PCR

The uterine tissues of mice and cells were lysed using TRIzol reagent (Thermo Fisher, USA) for total RNA extraction. Subsequently, cDNA was synthesized with the HiScript IV All-in-One Ultra RT SuperMix (Vazyme, China) according to the manufacturer’s instructions. qRT-PCR analysis was performed on a CFX96 Real-Time PCR System (Bio-Rad, Hercules, CA, USA) using the ChamQ Universal SYBR qPCR Master Mix (Vazyme, China) and appropriate primers. The reaction was carried out with a sample volume of 10 μL, initiating at 95 °C for 30 s, followed by 40 cycles of 95 °C for 5 s, 60 °C for 30 s, and 95 °C for 10 s. The mRNA expression levels of the target gene were normalized to GAPDH and calculated using the 2^−ΔΔCT^ method. The primer sequences utilized are detailed in supplementary Table S1.

### Western Blot Analysis

The total protein of uterine tissue of mice was extracted, and the concentration of protein was determined by using BCA Protein Assay kit (20201ES76, Yeasen Company, Shanghai, China). A total of 20 μg protein were added in each well and separated with 10% sodium dodecyl sulphate polyacrylamide gel electrophoresis for 1 h, and transferred onto a polyvinylidene fluoride membrane. The membrane was blocked with 5% skimmed milk, and then incubated with the following antibodies overnight at 4 °C: β-actin (66,009–1-Ig, 1:10,000) and FGF21 (ab171941, 1:1000). After washing with phosphate-buffered saline with Tween 20 (PBST) three times, the membrane was incubated with the HRP-labelled IgG secondary antibody for 1 h at room temperature, and then washed with PBST three times. Subsequently, the membrane was exposed in darkroom by using Electrochemiluminescence (ECL) solution (ECL808-25, Biomiga, San Diego, CA, USA), and then pressed. The relative expression of target protein was presented by the ratio of the grey level of target protein band to the β-actin band.

### Statistical Analysis

Statistical analyses were conducted by using GraphPad Prism 9 software. Measurement data were expressed as mean ± standard error of the mean (SEM). Comparison between two groups was performed by unpaired 2-tailed Student t test. Comparison among multiple groups was conducted by one-way ANOVA test. Results were expressed as percentage and analysed using chi-square test. P < 0.05 was considered significantly different.

## Results

### Macrophage-derived FGF21 is Positively Associated With Inflammatory Responses and Disease Progression in Endometritis

**Fig. 1:**
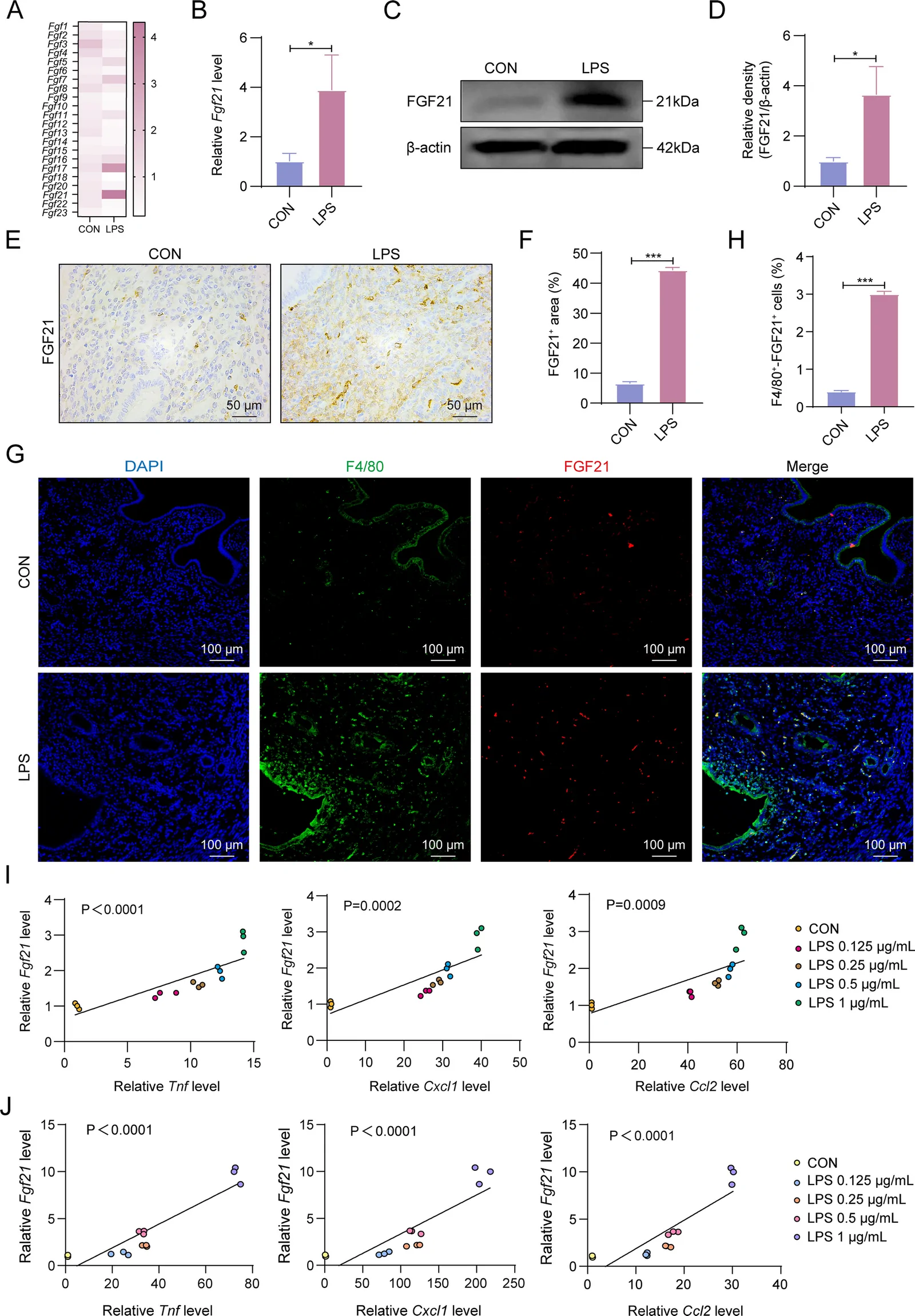
Macrophage-derived FGF21 is positively associated with inflammatory responses and disease progression in endometritis. (A) Heatmap showing the expression profile of the FGF family in control and LPS-induced endometritis uterine tissues. (B) qRT-PCR analysis of FGF21 mRNA expression in uterine tissues from wild-type mice under control and LPS-induced conditions. (C, D) Western blot analysis and quantification of FGF21 protein levels normalized to β-actin. (E, F) Immunohistochemical staining and quantification of FGF21 in uterine tissues. Scale bar: 50 μm. (G, H) Immunofluorescence staining showing co-localization of FGF21 (red) with the macrophage marker F4/80 (green), and corresponding quantification. Scale bar: 100 μm. (I) Correlation analysis between FGF21 expression and inflammatory cytokines (*Tnf*, *Cxcl1*, and *Ccl2*) in RAW264.7 cells stimulated with increasing concentrations of LPS (0.125–1 μg/mL). (J) Correlation analysis between FGF21 expression and inflammatory cytokines (*Tnf*, *Cxcl1*, and *Ccl2*) in BMDMs stimulated with increasing concentrations of LPS (0.125–1 μg/mL). Data are presented as mean ± SEM. *P < 0.05, **P < 0.01, ***P < 0.001

To systematically identify FGF family members involved in endometritis, we first profiled their expression in a lipopolysaccharide (LPS)-induced mouse model. Among all FGF members examined, FGF21 exhibited the most pronounced upregulation in inflamed uterine tissues (Fig. 1A). This observation was further validated by independent analyses, which confirmed that both FGF21 mRNA and protein levels were consistently elevated in LPS-treated uteri (Fig. 1B-D). Notably, immunohistochemical staining revealed not only a marked increase in FGF21 expression, but also a distinct cell type–specific distribution pattern within the inflamed endometrium (Fig. 1E-F). To determine the cellular source of FGF21, we performed immunofluorescence co-localization analysis and found that FGF21 was predominantly expressed in F4/80⁺ macrophages, with a substantial increase under inflammatory conditions (Fig. 1G-H). Given the central role of macrophages as key effector cells in endometritis, we next investigated whether FGF21 expression is associated with macrophage activation. Upon stimulation with graded concentrations of LPS, both RAW264.7 macrophages and primary BMDMs exhibited a dose-dependent inflammatory response, accompanied by a corresponding increase in FGF21 expression (Fig. 1I-J). Correlation analysis further demonstrated that FGF21 mRNA levels were positively associated with pro-inflammatory cytokines, including *Tnf*, *Cxcl1*, and *Ccl2* (Fig. 1I-J). Collectively, these results indicate that macrophage-derived FGF21 is closely linked to inflammatory activation and disease progression in endometritis, suggesting a potential role in modulating the uterine inflammatory microenvironment.

### Genetic Deletion of FGF21 Exacerbates the Severity of Endometritis

**Fig. 2:**
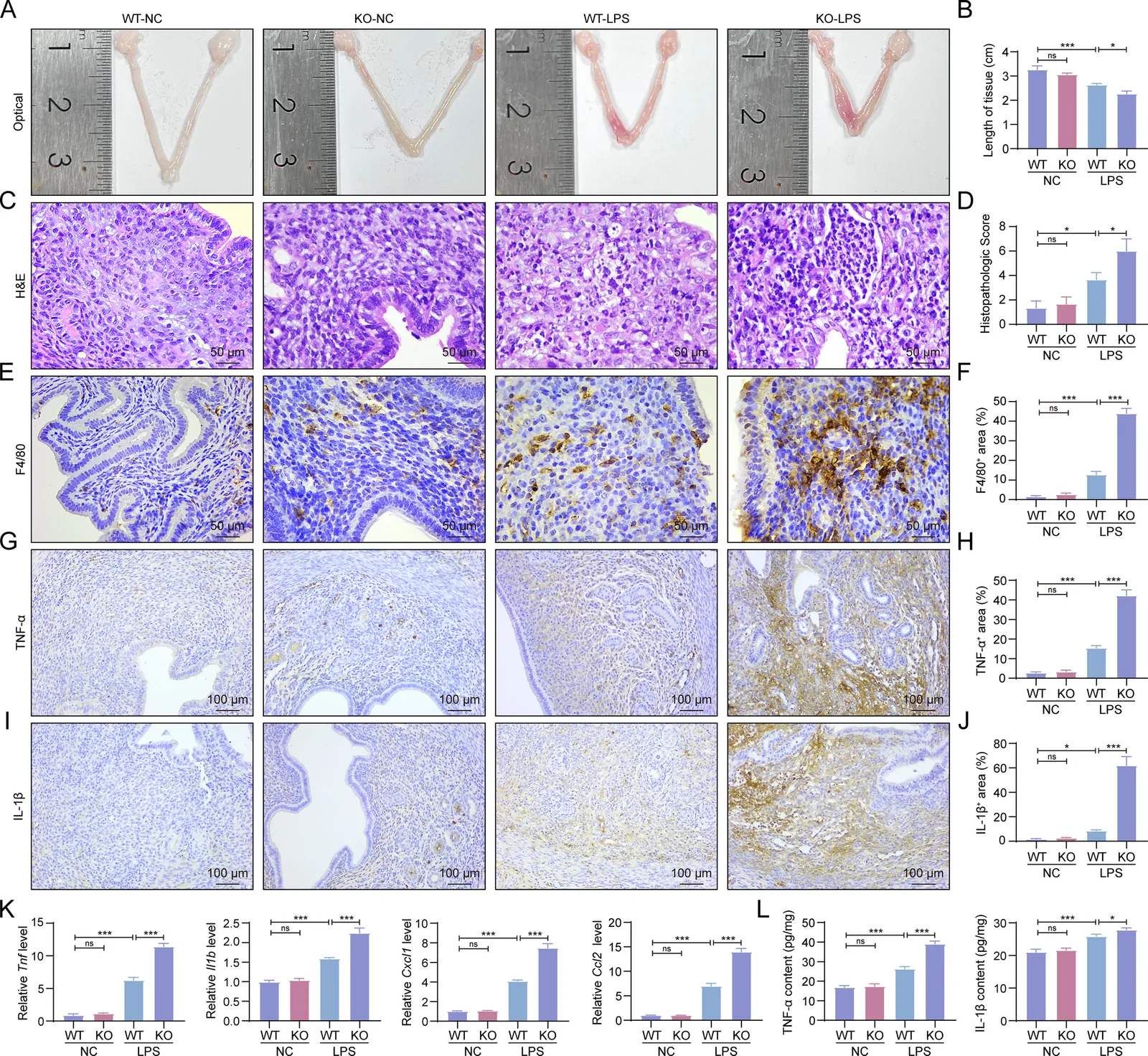
Genetic deletion of FGF21 exacerbates the severity of endometritis. (A) Representative gross morphology of uteri from wild-type and FGF21.^−/−^ mice under control and LPS-induced conditions. (B) Quantification of uterine length. (C, D) H&E staining and histopathologic scoring of uterine tissues. Scale bar: 50 μm. (E, F) Immunohistochemical staining and quantification of F4/80 in uterine tissues. (G, H) Immunohistochemical staining and quantification of TNF-α in uterine tissues. (I, J) Immunohistochemical staining and quantification of IL-1β in uterine tissues. (K) qRT-PCR analysis of inflammatory cytokines (*Tnf*, *Il1b*, *Cxcl1*, and *Ccl2*). (L) ELISA analysis of TNF-α and IL-1β levels in uterine tissues. Data are presented as mean ± SEM. *P < 0.05, **P < 0.01, ***P < 0.001

To determine the functional significance of FGF21 upregulation, we utilized FGF21 knockout (KO) mice. Following LPS challenge, FGF21 KO mice exhibited markedly aggravated pathological features compared with their wild-type (WT) counterparts. Macroscopically, uteri from KO mice displayed more severe swelling and congestion (Fig. 2A). Consistent with increased tissue edema, uterine length was significantly reduced under inflammatory conditions, with a more pronounced shortening observed in FGF21-deficient mice (Fig. 2B). Histological analysis showed that both WT and KO control mice maintained intact endometrial architecture. In contrast, LPS-treated WT mice exhibited typical pathological alterations, including disrupted tissue organization, expanded interstitial space, hemorrhage, epithelial damage, and neutrophil infiltration. Notably, these pathological changes were substantially exacerbated in FGF21 KO mice, which was further confirmed by significantly elevated histopathological scores (Fig. 2C-D). To further evaluate inflammatory responses, immunohistochemical staining was performed for the macrophage marker F4/80 and pro-inflammatory cytokines TNF-α and IL-1β. Compared with WT mice subjected to LPS, FGF21 KO mice exhibited markedly increased staining intensity for F4/80, TNF-α, and IL-1β in uterine tissues, indicating enhanced immune cell infiltration and inflammatory activation (Fig. 2E-J). At the molecular level, the mRNA expression of pro-inflammatory mediators, including *Tnf*, *Il1b*, *Cxcl1*, and *Ccl2*, was significantly elevated in FGF21 KO mice compared with WT mice following LPS treatment (Fig. 2K). Consistently, ELISA analysis confirmed increased protein levels of TNF-α and IL-1β in KO mice (Fig. 2L). Collectively, these results demonstrate that endogenous FGF21 plays a critical protective role in limiting inflammatory responses and tissue injury during endometritis.

### rhFGF21 Ameliorates Endometrial Injury and Suppresses Inflammation in LPS-induced Endometritis

**Fig. 3:**
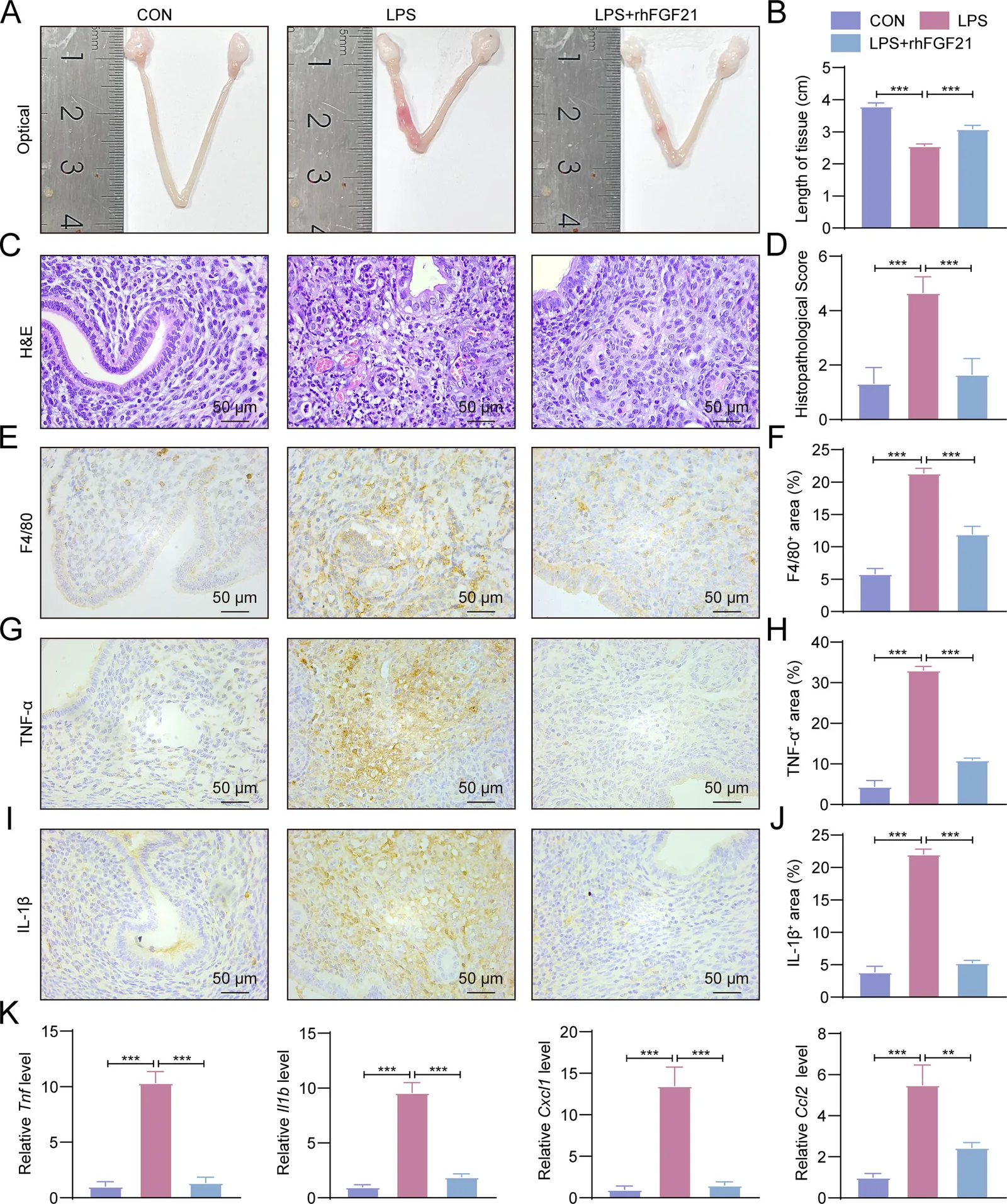
rhFGF21 ameliorates endometrial injury and suppresses inflammation in LPS-induced endometritis. (A) Representative gross morphology of uteri from control, LPS-treated, and LPS + rhFGF21-treated mice. (B) Quantification of uterine length. (C, D) H&E staining and histopathologic scoring of uterine tissues. Scale bar: 50 μm. (E, F) Immunohistochemical staining and quantification of F4/80 in uterine tissues. (G, H) Immunohistochemical staining and quantification of TNF-α in uterine tissues. (I, J) Immunohistochemical staining and quantification of IL-1β in uterine tissues. (K) qRT-PCR analysis of inflammatory cytokines (*Tnf*, *Il1b*, *Cxcl1*, and *Ccl2*). Data are presented as mean ± SEM. *P < 0.05, **P < 0.01, ***P < 0.001

We next evaluated the protective effect of exogenous FGF21 in endometritis by administering recombinant human FGF21 (rhFGF21) prior to intrauterine LPS challenge. Compared with LPS-treated mice, rhFGF21 administration markedly alleviated uterine edema and partially restored uterine length (Fig. 3A-B). Histological analysis further revealed improved tissue architecture and reduced inflammatory cell infiltration following rhFGF21 treatment, which was accompanied by a significant decrease in histopathological scores (Fig. 3C-D). Consistently, immunohistochemical staining showed that rhFGF21 attenuated LPS-induced macrophage infiltration, as indicated by reduced F4/80 positivity, along with decreased expression of pro-inflammatory cytokines TNF-α and IL-1β (Fig. 3E-J). Meanwhile, rhFGF21 significantly downregulated the mRNA expression of *Tnf*, *Il1b*, *Cxcl1*, and *Ccl2* (Fig. 3K). Together, these findings demonstrate that exogenous FGF21 effectively mitigates inflammatory responses and tissue injury in endometritis.

### FGF21 Attenuates Macrophage-mediated Inflammation and Promotes Endometrial Stromal Cell Survival and Repair

**Fig. 4:**
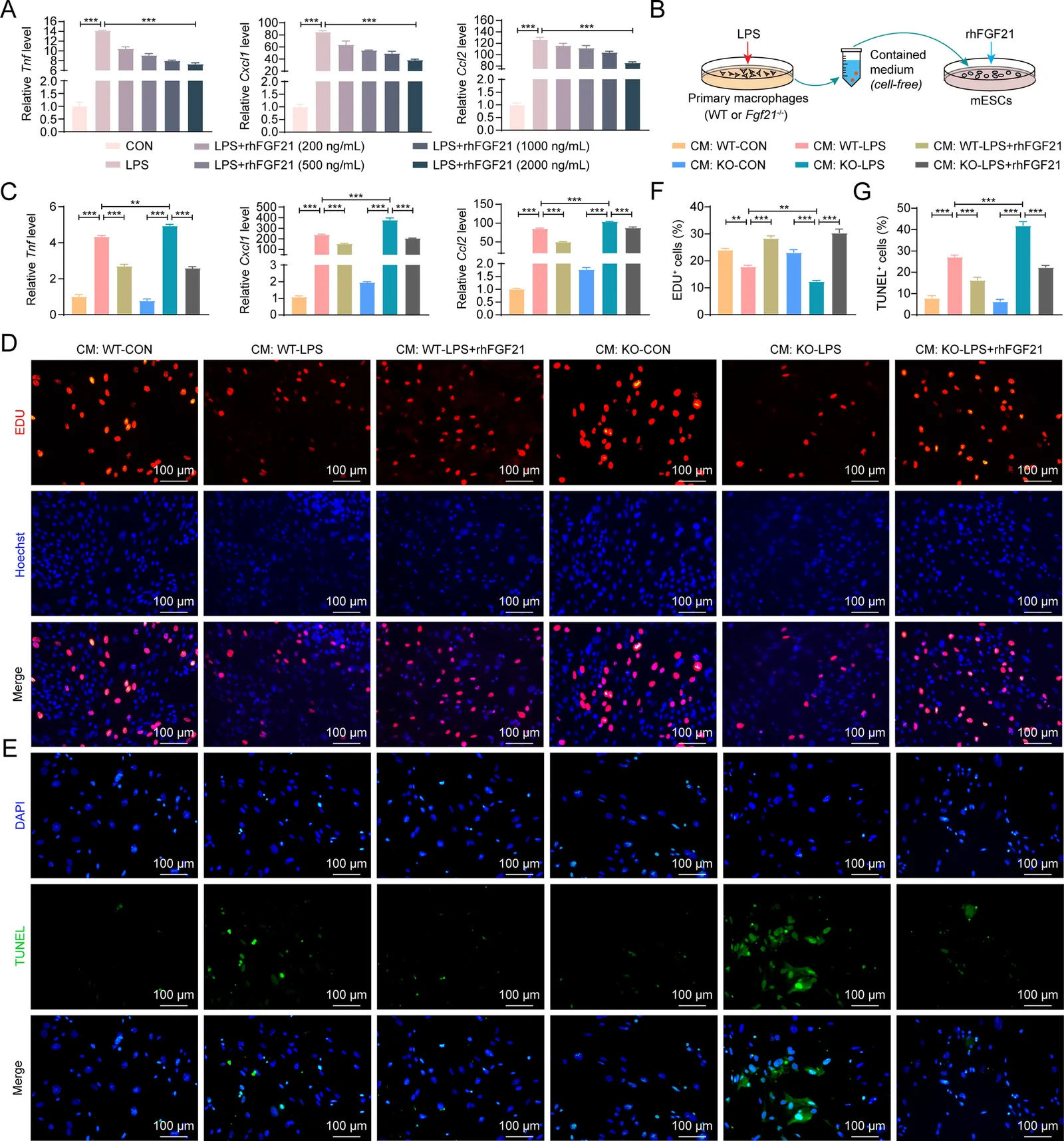
FGF21 suppresses macrophage-mediated inflammation and promotes proliferation while inhibiting apoptosis in mESCs. (A) qRT-PCR analysis of inflammatory cytokines (*Tnf*, *Cxcl1*, and *Ccl2*) in RAW264.7 cells treated with increasing concentrations of rhFGF21. (B) Schematic diagram of the macrophage–mESC co-culture system using conditioned medium derived from wild-type or FGF21.^−/−^ macrophages with or without LPS stimulation. (C) qRT-PCR analysis of inflammatory cytokines in mESCs under different treatment conditions. (D, E) Representative images of EdU and TUNEL staining showing proliferation and apoptosis in mESCs. Scale bar: 100 μm. (F, G) Quantification of EdU-positive and TUNEL-positive cells. Data are presented as mean ± SEM. *P < 0.05, **P < 0.01, ***P < 0.001

To further elucidate the cellular mechanisms underlying the protective effects of FGF21, we performed in vitro experiments focusing on macrophage-mediated inflammatory responses and intercellular communication within the endometrial microenvironment. In RAW264.7 macrophages, FGF21 treatment dose-dependently suppressed LPS-induced inflammatory activation (Fig. 4A). Consistently, rhFGF21 pretreatment significantly suppressed LPS‑induced inflammatory cytokine expression in primary BMDMs as well (Supplementary Figure S1). Given that FGF21 functions as a secreted cytokine, we next investigated its potential paracrine effects using an indirect co-culture system between macrophages and endometrial stromal cells (ESCs) or epithelial cells (EECs) (Fig. 4B). Conditioned media (CM) from LPS-stimulated macrophages markedly induced secondary inflammatory responses in ESCs, whereas CM derived from FGF21-deficient macrophages further exacerbated this effect, consistent with our in vivo observations. Notably, supplementation with recombinant human FGF21 attenuated ESCs inflammatory responses induced by both wild-type and FGF21-deficient macrophage-CM (Fig. 4C). In contrast, although macrophage-CM also triggered inflammatory responses in EECs, these effects were not significantly alleviated by FGF21 treatment, suggesting a cell type–specific responsiveness (Supplementary Figure S2A-B).

**Fig. 5:**
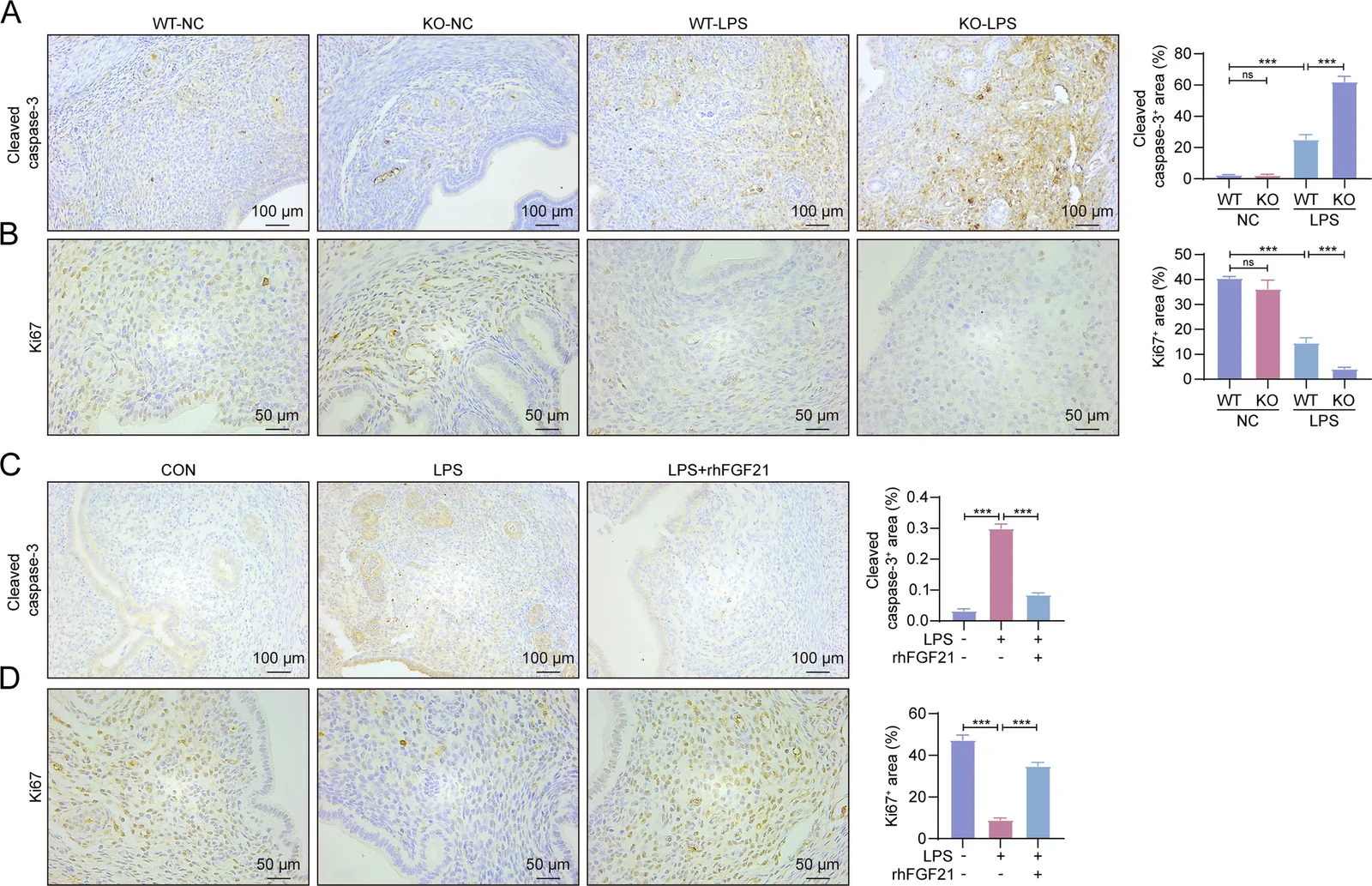
FGF21 regulates apoptosis and proliferation in vivo during endometritis. (A, B) Representative immunohistochemical staining and quantification of cleaved caspase-3 and Ki67 in uterine tissues from wild-type and FGF21.^−/−^ mice under control and LPS-induced conditions. (C, D) Effects of rhFGF21 treatment on pyroptosis and proliferation markers in LPS-induced endometritis. Scale bar: 50–100 μm. Data are presented as mean ± SEM. *P < 0.05, **P < 0.01, ***P < 0.001

Given the critical role of ESCs in maintaining endometrial structure and repair, we next assessed their proliferative and survival capacities. EdU and TUNEL assays revealed that CM from LPS-stimulated macrophages significantly inhibited ESC proliferation and increased cell death, with more pronounced effects observed in the FGF21-deficient group. Importantly, rhFGF21 supplementation partially restored ESC proliferation and reduced cell death (Fig. 4D-G). Consistent with these findings, in vivo analysis further demonstrated that LPS challenge increased cleaved caspase-3–positive cells and reduced Ki67-positive proliferating cells in uterine tissues, effects that were exacerbated in FGF21 KO mice but markedly reversed by rhFGF21 treatment (Fig. 5A-D). Notably, rhFGF21 treatment alone in healthy WT mice did not induce obvious changes in uterine morphology, H&E staining, F4/80, TNF-α, IL-1β, or cleaved caspase-3 expression compared with controls (Supplementary Figure S3A-C). Only Ki67 staining was mildly increased after rhFGF21 treatment (Supplementary Figure S3C), which is consistent with the known weak pro-proliferative activity of FGF21. Collectively, these results indicate that FGF21 mitigates endometritis not only by suppressing macrophage inflammatory activation but also by limiting macrophage-driven secondary injury and promoting ESCs survival and repair through paracrine mechanisms.

### The Therapeutic Efficacy of rhFGF21 Extends to MRSA-induced Infectious Endometritis

**Fig. 6:**
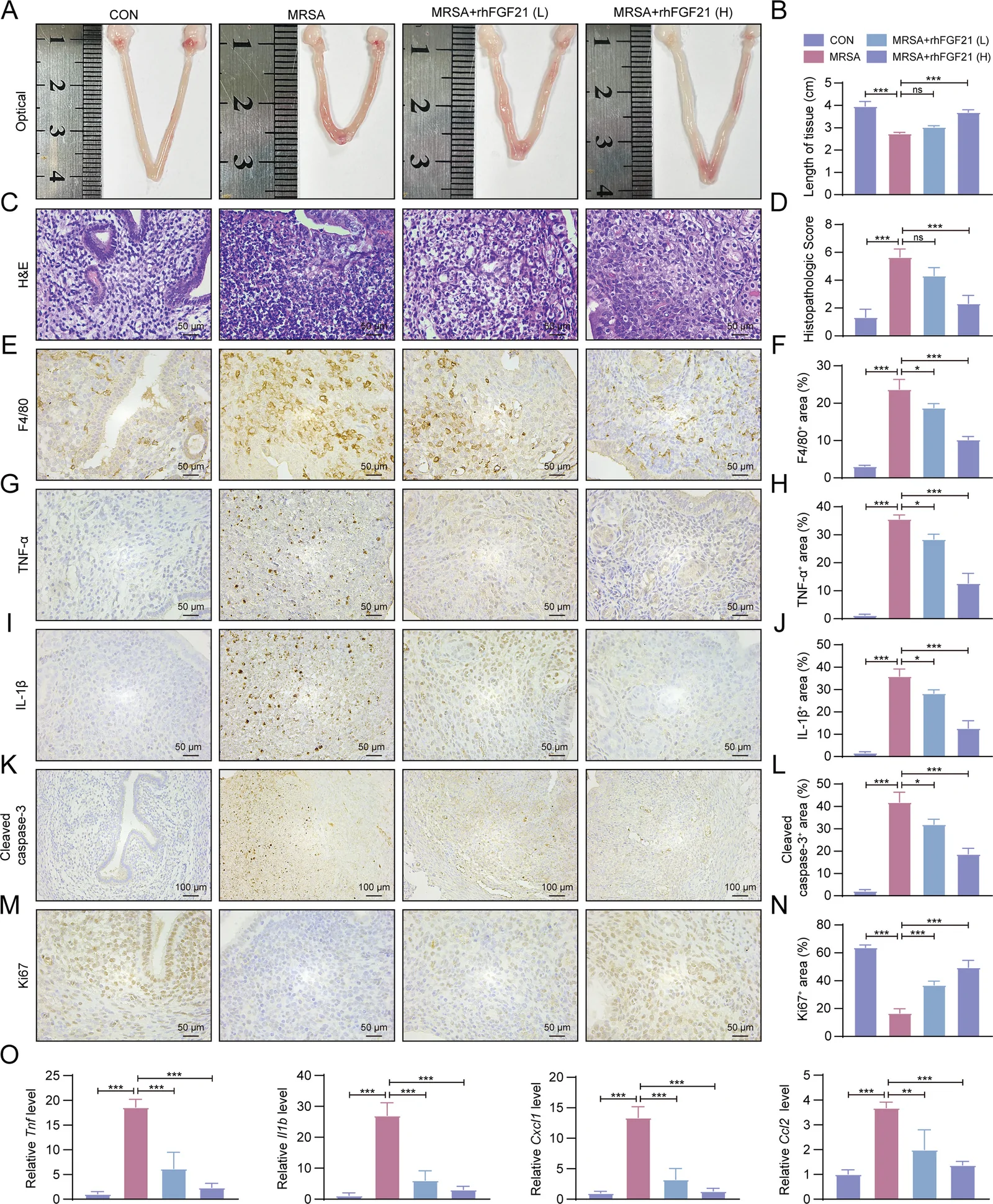
rhFGF21 dose-dependently alleviates MRSA-induced endometritis. (A) Representative gross morphology of uteri from control, MRSA-treated, and rhFGF21-treated groups at different doses (0.75 and 1.5 mg/kg). (B) Quantification of uterine length. (C, D) H&E staining and histopathologic scoring. Scale bar: 50 μm. (E, F) Immunohistochemical staining and quantification of F4/80 in uterine tissues. (G, H) Immunohistochemical staining and quantification of TNF-α in uterine tissues. (I, J) Immunohistochemical staining and quantification of IL-1β in uterine tissues. (K, L) Immunohistochemical staining and quantification of cleaved caspase-3 in uterine tissues. (M, N) Immunohistochemical staining and quantification of Ki67 in uterine tissues. (O) qRT-PCR analysis of inflammatory cytokines (*Tnf*, *Il1b*, *Cxcl1*, and *Ccl2*). Data are presented as mean ± SEM. *P < 0.05, **P < 0.01, ***P < 0.001

Building on the protective effects of FGF21 observed in LPS-induced endometritis, we next evaluated its therapeutic potential in a clinically relevant methicillin-resistant *Staphylococcus aureus* (MRSA)-induced model across different doses. MRSA infection induced pronounced uterine injury, as reflected by edema, tissue damage, and inflammatory infiltration, all of which were progressively alleviated by rhFGF21 treatment in a dose-dependent manner, with the greatest improvement observed at the high dose (Fig. 6A-D). Consistently, rhFGF21 reduced macrophage infiltration (F4/80⁺) and suppressed the expression of pro-inflammatory cytokines TNF-α and IL-1β in a dose-dependent manner (Fig. 6E-J). In addition to its anti-inflammatory effects, rhFGF21 also promoted tissue repair under infectious conditions. MRSA challenge markedly increased cleaved caspase-3–positive apoptotic cells and reduced Ki67-positive proliferating cells, whereas rhFGF21 treatment reversed these changes in a dose-dependent manner, decreasing apoptosis and restoring proliferative capacity (Fig. 6K-N). At the molecular level, rhFGF21 dose-dependently suppressed the expression of inflammatory mediators, including *Tnf*, *Il1b*, *Cxcl1*, and *Ccl2* (Fig. 6O). Collectively, these findings demonstrate that rhFGF21 exerts robust dose-dependent therapeutic effects in MRSA-induced endometritis, supporting its potential as a candidate intervention for infection-associated uterine inflammation.

### rhFGF21 Exerts Protective Effects Through Activation of FGFR1

**Fig. 7:**
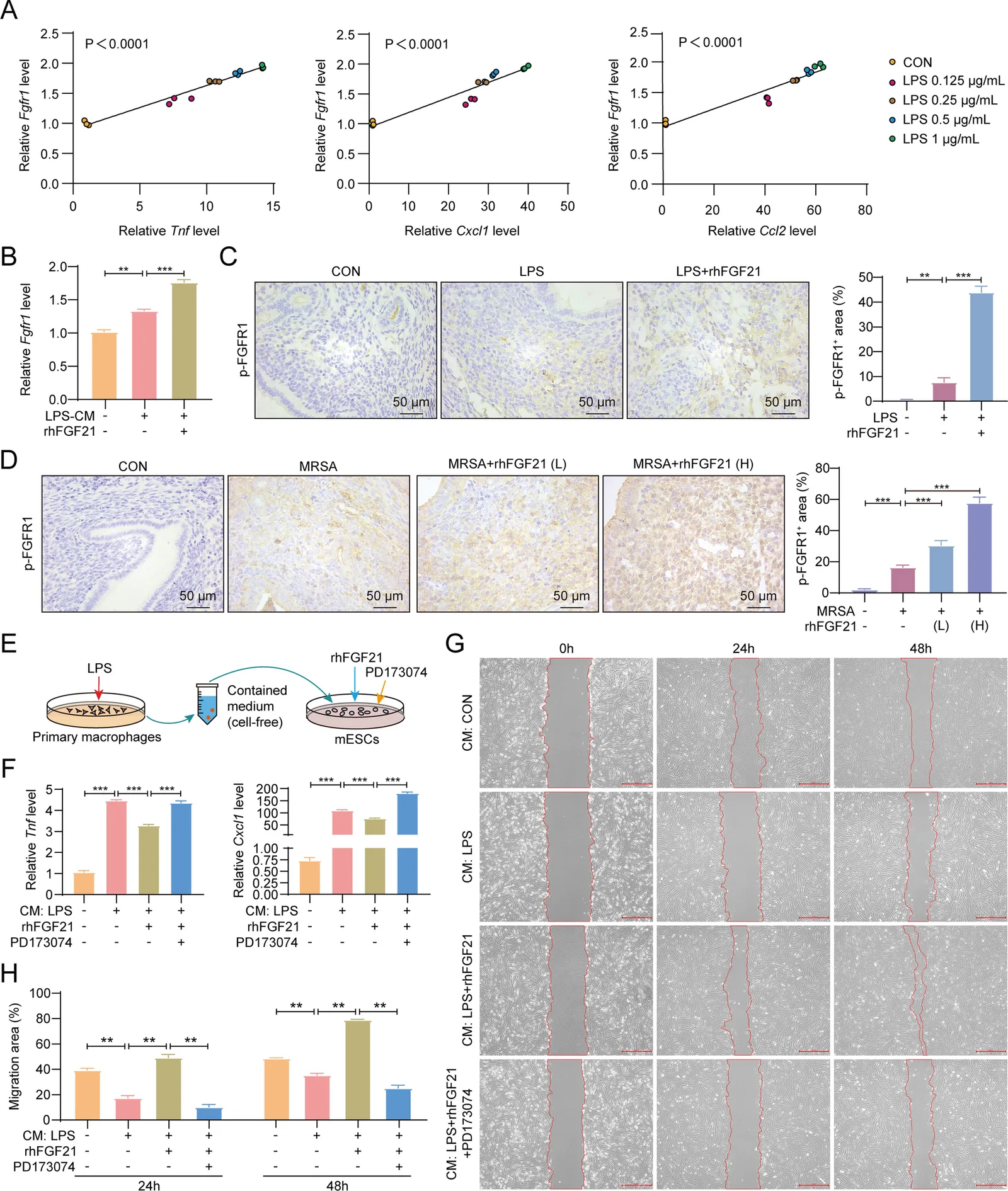
FGF21 exerts protection effects via FGFR1 activation. (A) Correlation analysis between *Fgfr1* and inflammatory cytokines (*Tnf*, *Cxcl1*, and *Ccl2*) in RAW264.7 cells treated with increasing concentrations of LPS. (B) qRT-PCR analysis of *Fgfr1* expression in mESCs treated with conditioned medium with or without rhFGF21. (C, D) Immunohistochemical staining and quantification of phosphorylated FGFR1 (p-FGFR1) in LPS- and MRSA-induced endometritis models. Scale bar: 50 μm. (E) Schematic diagram of the macrophage-mESC co-culture system with FGFR1 inhibition. (F) qRT-PCR analysis of inflammatory cytokines in mESCs under FGFR1 inhibition. (G, H) Scratch assay and quantification of cell migration under different treatment conditions. Scale bar: 500 μm. Data are presented as mean ± SEM. *P < 0.05, **P < 0.01, ***P < 0.001

To elucidate the molecular mechanism underlying the protective effects of FGF21 in endometritis, we focused on its canonical receptor, fibroblast growth factor receptor 1 (FGFR1) [32]. Consistent with previous reports that FGF21 signals through FGFR1, we observed that LPS stimulation significantly increased FGFR1 expression in RAW264.7 macrophages, paralleling the induction of pro-inflammatory cytokines (Fig. 7A), which is in line with the positive association between FGF21 and inflammatory mediators observed in Fig. 1I. In addition, ESCs exposed to CM from activated macrophages exhibited elevated FGFR1 expression, which was further enhanced upon rhFGF21 treatment, suggesting that FGF21 may act through FGFR1 to modulate inflammatory responses (Fig. 7B). We next assessed receptor activation and found that phosphorylation of FGFR1 (p-FGFR1) was markedly increased in both LPS- and MRSA-induced endometritis models. Notably, rhFGF21 administration further enhanced FGFR1 phosphorylation, indicating activation of FGFR1-dependent signaling pathways (Fig. 7C-D). To determine whether FGFR1 is required for the protective effects of FGF21, we employed the selective FGFR1 inhibitor PD173074 in the macrophage–ESC co-culture system (Fig. 7E). Pharmacological inhibition of FGFR1 abolished the anti-inflammatory effects of rhFGF21 in ESCs, as evidenced by restored inflammatory responses (Fig. 7F). Moreover, the well-established mild pro-proliferative activity of FGF21 enabled rhFGF21 to promote cell migration in wound-healing assays in both WT ESCs and LPS-injured ESCs (Fig. 7G-H, and Supplementary Figure S4). Notably, this protective pro-repair effect was markedly impaired following FGFR1 inhibition (Fig. 7G-H). Collectively, these results demonstrate that the protective effects of FGF21 in endometritis are mediated through activation of FGFR1 signaling, highlighting the FGF21–FGFR1 axis as a key regulatory pathway in controlling inflammation and tissue repair.

## Discussion

In this study, we systematically investigated the role of the fibroblast growth factor (FGF) family in endometritis and identified several key findings. First, FGF21 was markedly upregulated and positively associated with endometritis progression. Second, FGF21 exerted a protective role in endometritis by suppressing macrophage-mediated inflammation and promoting stromal cell proliferation and tissue repair. Third, exogenous administration of recombinant human FGF21 (rhFGF21) conferred significant therapeutic benefits through activation of FGFR1. Collectively, these findings establish FGF21 as a critical regulator in endometritis and support the potential of rhFGF21 as a therapeutic candidate.

A notable observation of this study is that FGF21 expression is robustly induced in uterine tissues during endometritis and is predominantly derived from infiltrating macrophages. This finding is particularly intriguing, as FGF21 has been traditionally characterized as a hepatokine, adipokine, or myokine, with high expression in metabolic organs such as liver, adipose tissue, and skeletal muscle [33–35]. Although its expression in immune cells has been reported, it is generally considered low and inducible under specific conditions [14]. Our results extend this paradigm by demonstrating that, within the inflammatory microenvironment of the uterus, macrophages become a major source of FGF21, suggesting a context-dependent reprogramming of FGF21 production and highlighting its previously unrecognized role as a local immunoregulatory factor.

Despite the positive correlation between FGF21 expression and disease severity, functional evidence from FGF21-deficient mice clearly demonstrated that loss of FGF21 exacerbates endometritis, indicating that its upregulation represents a compensatory protective response rather than a pathogenic driver. This pattern is consistent with observations in other pathological conditions, including obesity, metabolic-associated fatty liver disease [36], sepsis [14], and cardiovascular disorders [37], where FGF21 is similarly elevated but functions to mitigate inflammation, reduce tissue injury, and restore homeostasis. Together, these findings support a unifying model in which FGF21 acts as a stress-responsive hormone that is transcriptionally induced under inflammatory or metabolic stress to counteract disease progression.

Mechanistically, our study reveals a dual mode of action of FGF21 in the uterine microenvironment. On one hand, macrophage-derived FGF21 acts in an autocrine manner to restrain macrophage activation, thereby limiting the production of key pro-inflammatory cytokines such as TNF-α and IL-1β. On the other hand, FGF21 exerts paracrine effects on endometrial stromal cells through FGFR1-mediated pro-survival signaling, thereby maintaining stromal cell viability and regenerative capacity under inflammatory conditions. Accordingly, FGF21 deficiency exacerbates endometrial injury through two complementary mechanisms: enhanced macrophage hyperactivation with excessive release of pro-apoptotic cytokines, and loss of direct pro-survival signaling in stromal cells, rendering them more susceptible to inflammatory damage. Consistently, our co-culture experiments demonstrated that conditioned medium from FGF21-deficient macrophages induced exaggerated secondary inflammation, increased apoptosis, and impaired proliferative capacity in endometrial stromal cells, all of which were effectively reversed by exogenous rhFGF21 supplementation. Together, these findings identify FGF21 as a critical mediator of immune–stromal crosstalk that reshapes the uterine microenvironment toward inflammation resolution and tissue repair.

From a translational perspective, it is particularly noteworthy that the protective effects of rhFGF21 were consistently validated in a clinically relevant methicillin-resistant *Staphylococcus aureus* (MRSA)-induced model of infectious endometritis. This not only strengthens the therapeutic potential of FGF21 but also suggests that its beneficial effects are not limited to LPS-induced inflammation. Instead, FGF21 likely targets convergent downstream pathways shared by diverse pathogenic stimuli, such as Toll-like receptor signaling and inflammatory cascades, supporting its potential as a broad-spectrum anti-inflammatory and pro-repair therapeutic strategy.

At the mechanistic level, we further demonstrate that the protective effects of FGF21 are largely mediated through activation of FGFR1. Our data show that inflammatory stimuli upregulate FGFR1 expression both in vitro and in vivo, and that FGFR1 activation is essential for FGF21-driven anti-inflammatory and pro-repair responses. Pharmacological inhibition of FGFR1 using PD173074 effectively abolishes these effects, providing compelling evidence for a functional coupling between FGF21 and FGFR1 in the context of uterine inflammation. These findings establish the FGF21–FGFR1 axis as a central signaling pathway and a promising target for therapeutic intervention in endometritis.

Despite these advances, several limitations should be acknowledged. First, while our study establishes the essential role of FGFR1, the downstream intracellular signaling cascades remain incompletely defined and warrant further investigation. Second, our work focuses on acute models of endometritis; whether FGF21 exerts similar protective effects in chronic endometritis and how it influences long-term reproductive outcomes, such as fertility and embryo implantation, remain to be determined. Addressing these questions will be critical for translating these findings into clinical applications.

## Conclusion

This study expands the current understanding of FGF21 biology by uncovering its role as a macrophage-derived factor in the reproductive system. By simultaneously suppressing inflammation and promoting tissue repair, targeting the FGF21–FGFR1 axis represents a dual-functional therapeutic strategy with considerable potential for the treatment of endometritis and possibly other inflammatory diseases.

## Electronic Supplementary Material

Below is the link to the electronic supplementary material.

Supplementary file1 (DOCX 1.74 MB)

Supplementary file2 (DOCX 662 KB)

## Data Availability

No datasets were generated or analysed during the current study.
