# Supplementary material for: Macrophage-derived FGF21 Restrains Endometritis By Suppressing Inflammation and Promoting Repair Through FGFR1
Source: Inflammation. 2026 Jul 21;49(1):203. doi: 10.1007/s10753-026-02537-z (PMC13672570; doi:10.1007/s10753-026-02537-z)
Supplement: Supplementary file 1 [file 10753_2026_2537_MOESM1_ESM.docx]

**Macrophage-derived FGF21 restrains endometritis by suppressing inflammation and promoting repair through FGFR1**

Haiyan Liu^1,#^, Zhuo Shi^1,#^, Yihui Zheng^2^, Xianliang Chen^1^, Fatma Mrisho Haji ^1^, Weiwei Zhu^3,^*, Feng Lin^1,^*

**Affiliations:**

*^1^ Department of Gynecology, the First Affiliated Hospital of Wenzhou Medical University, Wenzhou, Zhejiang, China;*

*^2^ The Second Affiliated Hospital and Yuying Children's Hospital of Wenzhou Medical University, Wenzhou, Zhejiang, China;*

*^3^ Department of Cardiology, the First Affiliated Hospital of Wenzhou Medical University, Wenzhou, Zhejiang, China;*

*^#^ Contributed equally*

*** **Corresponding authors:**

Prof. Weiwei Zhu, Ph.D., at the First Affiliated Hospital, Wenzhou Medical University, Wenzhou, Zhejiang 325035, China.

E-mail: weiweizhu@wmu.edu.cn

and

Prof. Feng Lin, Ph.D., at the First Affiliated Hospital, Wenzhou Medical University, Wenzhou, Zhejiang 325035, China.

E-mail: lin801026@163.com

*Supplementary Information*

**Supplementary Figures**

**Fig. S1. FGF21 inhibits LPS-induced inflammatory responses in BMDMs.** qRT-PCR analysis of inflammatory cytokines (*Tnf*, *Cxcl1*, and *Ccl2*) in BMDMs treated with rhFGF21. All the data were presented as means ± SEM. *P < 0.05; **P < 0.01; ***P < 0.001.

**Fig. S2. FGF21 does not inhibit secondary inflammatory responses in mEECs.** (A) Schematic diagram of the macrophage–mEEC co-culture system using conditioned medium derived from wild-type or FGF21^-/-^ macrophages with or without LPS stimulation. (B) qRT-PCR analysis of inflammatory cytokines in mEECs under different treatment conditions. All the data were presented as means ± SEM. *P < 0.05; **P < 0.01; ***P < 0.001.

**Fig. S3. FGF21 alone exerts negligible effects on the physiologically normal uterus.** (A) Representative gross morphology of uteri from control and rhFGF21-treated groups and quantification of uterine length. (B) H&E staining and histopathologic scoring. Scale bar: 50 μm. (C) Immunohistochemical staining and quantification of F4/80, TNF-α, IL-1β, cleaved caspase-3, Ki67 in uterine tissues. All the data were presented as means ± SEM. *P < 0.05; **P < 0.01; ***P < 0.001.

**Fig. S4. FGF21 alone exerts mild pro-proliferative effects on physiologically normal mESCs.** Scratch assay and quantification of cell migration in control and rhFGF21‑alone groups. Scale bar: 500 μm. All the data were presented as means ± SEM. *P < 0.05; **P < 0.01; ***P < 0.001.

**Supplementary Tables**

**Table S1. Mouse primers used for real-time qPCR assay.**

| **Gene** | **Forward sequence** | **Reverse sequence** |
| --- | --- | --- |
| *Gapdh* | AGGTCGGTGTGAACGGATTTG | GGGGTCGTTGATGGCAACA |
| *Il1b* | GCAACTGTTCCTGAACTCAACT | ATCTTTTGGGGTCCGTCAACT |
| *Tnf* | CCCTCACACTCAGATCATCTTCT | GCTACGACGTGGGCTACAG |
| *Cxcl1* | CAAACCGAAGTCATAGCCAC | TGGGGACACCTTTTAGCATC |
| *Ccl2* | CAGCCAGATGCAATCAATGCC | TGGAATCCTGAACCCACTTCT |
| *Fgfr1* | ACCAACCTCTAACCGCAGAAC | GACTCCACTTCCACAGGGACT |
| *Fgf1* | CCCTGACCGAGAGGTTCAAC | GTCCCTTGTCCCATCCACG |
| *Fgf2* | GCGACCCACACGTCAAACTA | TCCCTTGATAGACACAACTCCTC |
| *Fgf3* | TGCGCTACCAAGTACCACC | CACCGCAGTAATCTCCAGGAT |
| *Fgf4* | TGCCTTTCTTTACCGACGAGT | GCGTAGGATTCGTAGGCGTT |
| *Fgf5* | AAGTAGCGCGACGTTTTCTTC | CTGGAAACTGCTATGTTCCGAG |
| *Fgf6* | CAGGCTCTCGTCTTCTTAGGC | TTCACACCCGAAATCTCTCCA |
| *Fgf7* | CTCTACAGGTCATGCTTCCACC | ACAGAACAGTCTTCTCACCCT |
| *Fgf8* | CCGAGGAGGGATCTAAGGAAC | CTTCCAAAAGTATCGGTCTCCC |
| *Fgf9* | ATGGCTCCCTTAGGTGAAGTT | TCATTTAGCAACACCGGACTG |
| *Fgf10* | TTTGGTGTCTTCGTTCCCTGT | TAGCTCCGCACATGCCTTC |
| *Fgf11* | TAGCCTGATCCGACAGAAGC | GGCAGAACAGTTTGGTGACG |
| *Fgf12* | CTCGGGGTGTTCAGCAAAGT | TTTCGTCCTTGGTCCCATCAA |
| *Fgf13* | AGAGTCCCCCTTTCGTGCTAA | GCTCTTCGGGTTCTGTGTTTTC |
| *Fgf14* | GGCTTGATCCGTCAGAAACG | CACCAGGTTGCCATTGAAAAG |
| *Fgf15* | GAAGACGATTGCCATCAAGGA | CGAATCAGCCCGTATATCTTGC |
| *Fgf16* | GTGTTTTCCGGGAACAGTTTGA | GGTGAGCCGTCTTTATTCAGG |
| *Fgf17* | GGCAGAGAGCGAGAAGTACAT | CGGTGAACACGCAGTCTTTG |
| *Fgf18* | CCTGCACTTGCCTGTGTTTAC | TGCTTCCGACTCACATCATCT |
| *Fgf20* | AGGATCACAGTCTCTTCGGTATC | GTCATTCATCCCAAGGTACAGG |
| *Fgf21* | GTGTCAAAGCCTCTAGGTTTCTT | GGTACACATTGTAACCGTCCTC |
| *Fgf22* | CCAGGACAGTATAGTGGAGATCC | AGTAGACCCGCGACCCATAG |
| *Fgf23* | CTCTACAGGTCATGCTTCCACC | AGCCAAGCAATGGGGAAGTG |
