# Supplementary material for: Macrophage-derived FGF21 Restrains Endometritis By Suppressing Inflammation and Promoting Repair Through FGFR1
Source: Inflammation. 2026 Jul 21;49(1):203. doi: 10.1007/s10753-026-02537-z (PMC13672570; doi:10.1007/s10753-026-02537-z)
Supplement: Supplementary file 2 [file 10753_2026_2537_MOESM2_ESM.docx]

**Macrophage-derived FGF21 restrains endometritis by suppressing inflammation and promoting repair through FGFR1**

Haiyan Liu^1,#^, Zhuo Shi^1,#^, Yihui Zheng^2^, Xianliang Chen^1^, Fatma Mrisho Haji^1^, Weiwei Zhu^3,^*, Feng Lin^1,^*

**Affiliations:**

*^1^ Department of Gynecology, the First Affiliated Hospital of Wenzhou Medical University, Wenzhou, Zhejiang, China;*

*^2^ The Second Affiliated Hospital and Yuying Children's Hospital of Wenzhou Medical University, Wenzhou, Zhejiang, China;*

*^3^ Department of Cardiology, the First Affiliated Hospital of Wenzhou Medical University, Wenzhou, Zhejiang, China;*

*^#^ Contributed equally*

*** **Corresponding authors:**

Prof. Weiwei Zhu, Ph.D., at the First Affiliated Hospital, Wenzhou Medical University, Wenzhou, Zhejiang 325035, China.

E-mail: weiweizhu@wmu.edu.cn

and

Prof. Feng Lin, Ph.D., at the First Affiliated Hospital, Wenzhou Medical University, Wenzhou, Zhejiang 325035, China.

E-mail: lin801026@163.com

**Blots images**

50kDa

70kDa

40kDa

35kDa

β-actin

CON LPS

10kDa

15kDa

20kDa

25kDa

FGF21

CON LPS

β-actin

50kDa

70kDa

40kDa

35kDa

CON LPS

CON LPS

25kDa

CON LPS

CON LPS

CON LPS

10kDa

15kDa

20kDa

CON LPS

FGF21
